# Integrated Pest Management of Wireworms in Potatoes: Use of Tolerant Varieties to Implement Damage Prevention

**DOI:** 10.3390/insects16010004

**Published:** 2024-12-26

**Authors:** Furlan Lorenzo, Bona Stefano, Benvegnù Isadora, Cacitti Valentina, Govoni Fausto, Parisi Bruno

**Affiliations:** 1 Veneto Agricoltura, 35020 Legnaro, Italy; isadora.benvegnu@gmail.com; 2 Department of Agronomy, Food, Natural Resources, Animals and Environment, University of Padova, 35020 Legnaro, Italy; stefano.bona@unipd.it; 3 Phytosanitary and Chemical Service, Research, Experimentation and Technical Assistance, Regional Agency for Rural Development—ERSA FVG, 33050 Pozzuolo del Friuli, Italy; valentina.cacitti@ersa.fvg.it; 4 Council for Agricultural Research and Economics (CREA)—Research Centre for Plant Protection and Certification (CREA-DC), 40128 Bologna, Italy; fausto.govoni@crea.gov.it; 5 Council for Agricultural Research and Economics (CREA)—Research Centre for Cereal and Industrial Crops (CREA-CI), 40128 Bologna, Italy; bruno.parisi@crea.gov.it

**Keywords:** *Agriotes*, *Agriotes sordidus*, *Solanum tuberosum*, 4x-breeding clones, glycoalkaloids, phenolic compounds

## Abstract

Wireworms (*Agriotes* spp., Coleoptera, Elateridae) are a major threat to potatoes, as are the current commercial standards for assessing potato damage. To reduce wireworm impacts on potato crops and comply with IPM legislation, we started research to assess the potential for new Italian 4x-breeding clones to reduce wireworm feeding on daughter tubers. Two sets of trials were carried out over a six-year period (2018–2023): in-field and in semi-natural conditions, with pots used to introduce a set number of reared wireworms. In the field trials, the varieties were planted in 4.8 × 9–12 m plots in a randomized-block layout with at least three replications. The same wireworm damage assessment was used for both sets of trials. The assessment involved counting all the erosions/scars caused by wireworm feeding activity. The prevalent wireworm species studied was *Agriotes sordidus*. Both sets of trials showed that some 4x-breeding clones were tolerant to wireworm attacks. The percentages of tubers damaged (any symptom) or with at least one ordinary/large hole was up to five times lower than in the commercial varieties. Glycoalkaloids and the caffeic acid content in tubers are considered to be the main cause of lower appetibility to wireworms.

## Simple Summary

The present work assesses the potential for new Italian 4x-potato breeding clones to reduce wireworm damage. Two sets of trials were carried out over a six-year period (2018–2023): in-field and in semi-natural conditions, with pots used to introduce a set number of reared wireworms. The same wireworm damage assessment was used for both sets of trials. The assessment involved counting all the erosions/scars caused by wireworm feeding activity. The prevalent wireworm species studied was *Agriotes sordidus*. Both sets of trials showed that some genotypes were tolerant to wireworm attacks. The percentage of tubers damaged was up to five times lower than in the commercial varieties. Tubers naturally contain glycoalkaloids and caffeic acid, a phenolic compound considered beneficial to human health; its high concentrations, however, are considered to be the main causes of lower appetibility to wireworms. Our research demonstrated that new potato genotypes with low wireworm damage are available for supply chains, benefitting both farmers and consumers. This achievement was possible without using synthetic insecticides, thus avoiding an undesirable impact on the environment and human beings.

## 1. Introduction

Wireworms (Coleoptera, Elateridae) are a phytosanitary problem worldwide [1]. According to FAOSTAT data [2], EU-27-cultivated potatoes (*Solanum tuberosum* L.) were harvested on 1.4 million hectares in 2022. Current European regulations on commercial quality for early and ware potatoes [3] can significantly increase scrap when they are affected by wireworm damage. Indeed, larvae holes and tunnels can compromise tuber appearance (early and ware potatoes) and lower the market value, resulting in additional costs for potato growers and packers, e.g., sorting, as well as in downgrading or rejection of affected lots.

Concern about EU potato wireworm damage has increased in recent years due to a general rise in damage at harvesting [4]. Although EU legislation on the sustainable use of pesticides was issued in 2009 (Directive 2009/128/CE), a general failure by potato growers to implement Integrated Pest Management (IPM) principles is considered to be the main cause of rising wireworm damage [5]. As potato crops had always been protected mainly by synthetic insecticides [6], when the most effective persistent ones were phased out due to their severe environmental impact [7,8], potato growers were completely unprepared to deal with soil pests. Phenylpyrazole fipronil and neonicotinoid thiamethoxam and the organophosphates ethoprophos and chlorpyrifos were the main synthetic insecticides to be banned (in 2014, 2018, 2019, and 2020 respectively). Today, it is therefore more important than ever to exploit IPM, as it massively reduces synthetic active substances. IPM principles are based on extensive field experiments and scientific research [9], becoming compulsory across the EU with Directive 2009/128/CE. The three main principles are as follows: prevention, i.e., keep harmful organism populations low, thus reducing the need for pest control, with strategies, including raising agroecosystem complexity/stability with appropriate rotations, implementing agronomic solutions, and using resistant/tolerant varieties and lower risk planting/harvesting dates; monitoring, i.e., use of sampling/models/thresholds, with synthetic plant protection products applied only when damage thresholds are exceeded, meaning that the prophylactic use of any chemical pesticide is unacceptable; and the replacement of synthetic insecticides with sustainable biological tools or other non-chemical strategies. Synthetic chemical pesticides are a last resort once all non-chemical practices are found to be unsuitable or have failed [9]. Implementing IPM successfully also involves supporting Insecticide Resistance Management (IRM), which prevents or delays pests developing insecticide resistance or helps resistant populations regain susceptibility.

The tolerance of potato cultivars to wireworm damage can be a strategic management tool, with scientific research being reviewed by Andrews et al. [10]. In general, the flesh and skin of potato cultivars contain various secondary plant metabolites, such as antioxidants and phenolic compounds, that can affect both the nutritional value and possible tolerances in the field [11]. Fasulati et al. [12] further showed that potato tolerance to phytopathogens is crucial because pathogens and their metabolites affect tuber attractiveness to wireworms. In Scotland, Johnson et al. [13] found significant differences in susceptibility to wireworm damage caused by *Agriotes obscurus* L. and *A. lineatus* L. between various in-the-field cultivars. The least susceptible (Bionica) had significantly greater concentrations of the glycoalkaloids (α-solanine and α-chaconine) than the other cultivars, while King Edwards had significantly greater concentrations of α-chaconine than the two most susceptible: Marfona and Maris Peer [13].

The susceptibility of some potato cultivars to *A. obscurus* feeding is known to be related to the total glycoalkaloid (TGA) content in daughter tubers [14,15]. Yencho et al. [16] found a clear link between variety tolerance to damage by the Colorado Potato Beetle (*Leptinotarsa decemlineata* Say, CPB) and only one glycoalkaloid molecule group (leptine). Glycoalkaloids (GAs) are bioactive molecules identified as natural insecticides [17], playing a role in both a plant’s constitutive and induced systemic resistance [18,19]. GAs, however, are also antinutritional and toxic to humans, with regulations limiting the amounts of TGAs allowed in candidate potato varieties. The TGA concentration of commercial potatoes rarely exceeds 100 mg/kg fresh weight [20,21]. The widely accepted safety limit for TGA levels in tubers is 200 mg/kg fresh weight [22]. However, the German Federal Institute for Risk Assessment (BfR) recently indicated 0.5 mg GAs/kg body weight as the No Observed Adverse Effect Level (NOAEL), recommending the safety limit be brought below 100 mg/kg fresh weight [23]. Although tubers contain these unbeneficial GAs, their periderm is a good source of phenolic compounds [24,25], health-related phytonutrients with antioxidant properties, which combat degenerative diseases [26].

It is only recently that potatoes have been bred specifically to resist wireworms [27]. Germplasms from wild potato relatives from South America (*Solanum berthaultii* Hawkes and *S. etuberosum* Lindl.) were crossed with a commercial potato variety, producing tolerant breeding clones that showed wireworm damage equal to that observed in insecticide-treated crops [28]. Some of these tolerant breeding clones contain TGA levels suitable for human consumption, which suggests that they could be used to develop wireworm-tolerant commercial varieties in the future.

Wild potato species are often invaluable sources of resistance traits that can be incorporated into cultivated potatoes, but incompatibility barriers often hamper backcrosses with *S. tuberosum*, making the introgression of resistance from wild relatives into new potato varieties very difficult [29]. In the early 1980s, Cornell University used *S. berthaultii* as a donor in a long-term breeding program [30] designed to raise resistance to the CPB. It resulted in the 4x-breeding clone Q 115-6, which was selected for its resistance to the Potato Tuber Moth (*Phthorimaea operculella* Zeller, PTM) by the Instituto de Investigaciones Agropecuarias (INIA), Chile [31,32]. This is one of the few clones with improved resistance to herbivore insects derived from a breeding program.

Subsequently, Italy’s Research Centre for Cereals and Industrial Crops (CREA-CI) obtained thirteen advanced 4x-breeding clones by backcrossing Q 115-6 with several potato cultivars chosen for their valuable traits, such as tolerance/resistance to Late Blight (*Phytophthora infestans* (Mont.) de Bary, LB) and Potato Cyst Nematodes (PCN), with some clones showing a good range of PTM tolerance [33]. Their tolerance was attributed to the 4x-breeding clones causing mortality during the early stages of larval development, suggesting the following:

- The α-chaconine and caffeic acid content under the periderm could play a defensive role against PTM;
- The PTM-tolerant 4x-breeding clone ISCI 181/10-4, which has high levels of these bio-compounds and a conspicuous phenolic content, may be useful in future breeding programs designed to defend plants and enhance nutritional value.

This paper intends to demonstrate the following:

- That new 4x-breeding clones showing PTM tolerance may reduce wireworm damage as well;
- That wireworm-tolerant genotypes with good agronomic potential can be used to implement IPM packages immediately and prevent significant wireworm damage, mainly by exploiting IPM Principle 1 (prevention with tolerant varieties), without synthetic insecticides being applied.

## 2. Materials and Methods

### 2.1. Pot Experiments

An experiment in semi-natural conditions was conducted between 31 March, 2022, and 5 May 2022, in a glass greenhouse at the University of Padova’s “Lucio Toniolo” experimental farm, Legnaro, Italy (coordinates 45.35934546086371, 11.943106988402056). The experiment was set up as in Civolani et al. 2021 [34] using a randomized-block design with six blocks, in which each one was a homogeneous replication that included all the treatments, i.e., one tuber of each variety (see Table 1). The substrate used was a mix of 70% loam collected in untreated local fields and 30% river sand kept at maximum water capacity. The containers were 11.5 cm-high plastic pots with a 10 cm top diameter and 1.1 L volume; holes in the pots’ bases were plugged with cotton tissue, which prevented larvae from escaping and allowed excess water to drain. A 3–4 cm layer of soil was laid in the pot bottom, the tuber was placed upon it, and the pot was filled with another 3–4 cm of soil. The entire tuber surface was covered by soil. The potato genotypes listed in Table 1 were assessed. The tubers were carefully selected to ensure that each genotype comprised similar shapes and sizes (about 30–35 mm); one tuber was used per pot.

**Figure 1:**
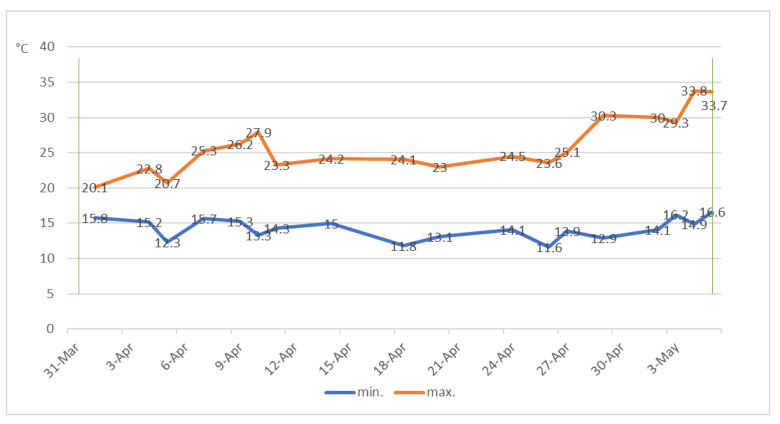
Temperature patterns during pot trials. Green vertical lines indicate pot preparation and the end of the trial.

The larvae had been produced in rearing cages over the previous two years with the method described by Furlan [35]. They were collected from the cages using bait traps, identified to species level, and sorted into different instars using size classes as described by Furlan [35]. Larvae were selected from the 7th–8th instar and in the active feeding phase [35]. Immediately after the pots had been filled with soil at water capacity and one tuber placed in the center, 2 cm deep, six *Agriotes sordidus* Illiger were added to each pot in Blocks 1, 2, and 3, and six *A. litigiosus* Rossi were added to each pot in Blocks 4, 5, and 6. An equal number of larvae-free pots were set up as a control; they were prepared in the same way as their “with larvae” peers. Each pot was inspected to collect data after six weeks. Temperature patterns are displayed in Figure 1.

#### 2.1.1. Potato Inspections and Surveys

The tubers were inspected every week, with rotten tubers replaced with new ones when appropriate. After four weeks, the tubers were removed from their pots for a final evaluation of wireworm erosion. Pot contents were then turned out onto a towel, the soil was removed by hand, and the larvae found and divided into three groups:

1. Alive and moving (left on the towel and moving away quickly);
2. Dying (on the towel for a minute without moving in a specific direction), or almost immobile but alive;
3. Dead.

Missing larvae were calculated based on the difference.

Parameters were collected for every potato tuber, including the following:

- Number of superficial scars/holes;
- Number of deep scars/holes.

Each scar/hole was categorized as described in Table 2.

When an erosion was superficial (1–3 mm deep), it was considered “small”, regardless of its width.

### 2.2. Field Experiments

Fourteen genotypes were inspected over a six-year period at different sites in Northern Italy: six commercial cultivars (Agata, Monalisa, Monique, Primura, Morene, and Vivaldi) chosen according to their local adaptations; one 4x-breeding clone (ISCI 133/12-7) derived from a CS8617 × Innovator crossing; three 4x-breeding clones (ISCI 181/10-3, ISCI 181/10-4, ISCI 201/10-1) derived from a Bionica × Q 115-6 crossing; one 4x-breeding clone (ISCI 232/12-1) derived from a MN 99/03 × Q 115-6 crossing; one 4x-breeding clone (ISCI 207/11-2) derived from a Romanze × Q 115-6 crossing; and two related parents (Bionica and Q 115-6). Each year, the genotypes were planted in their own 4.8 × 9–12 m plot in a randomized block design with three replications.

The potato tubers were harvested at senescence growth stage BBCH 97 907, i.e., leaves and stem dead, stems bleached and dry. For each genotype, at least 100 U.S. N.1 tubers (5 cm diameter, or weighing a minimum 112 g) were selected from the four internal rows of each plot.

#### 2.2.1. Cultivation

Local agronomic practices were used on all experimental field-trial plots homogeneously at each site (Table 3 and Table S1). The sites were located a few meters above sea level, apart from Madesimo (1560 m asl). No soil synthetic insecticide was applied. The experimental field-trial plots were laid out in randomized blocks with at least three replications. The main characteristics of the field trials are summarized in Table 3.

#### 2.2.2. Assessment of Wireworm Species/Density

In some fields, bait traps [36,37] were deployed to assess wireworm populations in the spring or fall before planting. A total of eighteen traps were buried in each field trial in Asigliano 2022 and Budrio 2023, as per Furlan [37]. In all fields, the tuber samples taken for the wireworm damage assessment were inspected for larvae. Any wireworms found were counted and identified.

#### 2.2.3. Estimation of Soil-Pest Damage to Potatoes

See the description of pot trials (Section 2.1.1).

### 2.3. Larvae Identification

All the larvae used in the pot trials and found in the field trials were identified with a specific key [38].

### 2.4. Statistical Analysis

Data management and the initial analysis were performed using Microsoft Excel [39]. Additional analyses and visualizations were conducted in R [40] using the following packages: readxl [41]; ggplot2 [42]; dplyr [43]; ggpubr [44]; MASS [45]; car [46]; multcomp [47]; lme4 [48]; and Matrix [49].

Data normality was assessed using the Shapiro–Wilk test [50] and added to the basic Stats package in R [40]. Since the data available did not have Gaussian distributions, the following procedure was performed on all data. The data were analyzed with ANOVA after values had been transformed into ranks [51,52]. Rank means were separated with the Tukey HSD test (*p* < 0.05). All data were processed in R [40]. The data reported in Table 4, Table 5, Table 6, Table 7, Table 8 and Table 9 are the medians (percentage of wireworm attacks and percentage of ordinary and large erosions) of the sampled values.

The data in the last Table were gathered with the following procedure. Each trial location was ranked by performance, i.e., percentage of wireworm attacks. Given that not all varieties had been planted at every location, the median ranking for each genotype was calculated, and these values were then transformed into ranks. Therefore, the ranks presented are the median ranking positions that each genotype achieved. The same procedure was applied to calculate the percentage of ordinary and large erosions (second column). To obtain the last column, the median of the previous two columns (percentage of wireworm erosion and percentage of ordinary and large erosions) was calculated and then transformed into ranks. The colors range from green (best performance) to red (worst). The numbers of plots and locations are important because they indicate how reliable the data were. The higher the numbers, the more reliable the data.

## 3. Results

### 3.1. Pot Trials

Results of the pot trials are shown in Table 4, with observations on sampled potato varieties.

The ISCI 4x-breeding clones generally displayed lower median values for both total and severe erosions than most of the commercial varieties. Indeed, their median values for severe erosions, especially ISCI 181/10-3, ISCI 207/11-2 and their donor male parent Q 115-6, are statistically similar to *S. chacoense* #GLKS30919#, a wild species well-known for its resistance to wireworm attacks.

ISCI 181/10-3, Q 115-6 and ISCI 207/11-2 have a very low probability of “Total erosions” (0.82% to 3.32%) and “Severe damage” (1.48% to 3.72%), indicating notable differences with Colomba. ISCI 201/10-1 and ISCI 181/10-4 have a higher probability of “Total erosions” (24.92% and 32.06% respectively) and “Severe damage” (20.90% and 35.42% respectively), indicating they are more similar to Colomba than the other ISCI 4x-breeding clones. They are, however, still more likely to be different from Colomba, one of Italy’s most cultivated varieties, than the commercial varieties.

Note that the results for *S. chacoense* #GLKS30919# do not differ significantly from the top four ISCI genotypes, suggesting that they share high tolerance to wireworms. In contrast, the commercial varieties Sensation, Monalisa, Ambra, Vivaldi, and JB 007 have a very high probability (98.25% to 99.98%) of “Total erosions” and “Severe damage”, suggesting that they are very similar to Colomba. None of the varieties had any impact on wireworm survival (Table 6).

### 3.2. Field Experiments

#### Assessment of Wireworm Species/Density

At all the experimental sites, the prevalent species was *Agriotes sordidus* apart from in Friuli-Venezia Giulia where the specimens found belonged to *A. brevis* Càndeze. After the Friuli-Venezia Giulia trial, *A. brevis* larvae were found using the bait traps in the nearby cultivated fields (Cacitti, personal communication).

The ISCI 4x-breeding clones (ISCI 181/10-3, ISCI 181/10-4, ISCI 201/10-1, and ISCI 133/12-7) showed negligible (Budrio 2019) or even no wireworm damage (Madesimo 2023) when wireworm pressure was low. Wireworm total and severe damage rates were up to five-to-ten times lower in the donor male parent Q 115-6 and some new genotypes (ISCI 181/10-3, ISCI 181/10-4, and ISCI 201/10-1) than in the most susceptible commercial varieties when wireworm population levels were higher (Table 8 and Table 9). Both ISCI 181/10-3 and ISCI 201/10-1 have shown low levels of total and severe damage in multiple regions and years.

The reduction in the percentage of tubers with any or the most severe erosion was much greater and statistically significant in the field trials, with the three ISCI genotypes 181/10-3, 181/10-4 and 201/10-1 suffering very little wireworm damage. The same breeding clones that proved to be PTM tolerant were also found to be wireworm tolerant. The authors found a significant positive association between the α-chaconine and caffeic acid content under periderm and PTM larval mortality [33]. This makes it likely that these secondary plant metabolites can reduce damage by both Lepidoptera (PTM) and Coleoptera pests (wireworms).

## 4. Discussion

Although few significant differences emerged at *p* = 0.05, the pot trials found that some potato genotypes were more susceptible than others to wireworm attack, confirming previous studies [15,53,54].

Table 5 shows each genotype’s probability of wireworm attack and allows it to be compared with Colomba, the most susceptible one. Current commercially available tuber-seed varieties, particularly Colomba, Monalisa, Monique, and Vivaldi, were found to be the most susceptible. Colomba also showed the highest number of different sized holes. The susceptibility of these four varieties suggests that potato growers need to introduce more control measures to manage wireworms in the field.

More and greater significant differences emerged at *p* = 0.05 in the field trials, which may be explained by the two different sets of experimental conditions. The pot trial was a “no choice”, i.e., the wireworms selected were extremely eager to feed, but only one small potato was available. In the field trials, the wireworms, which are very polyphagous [55,56,57], had alternatives to daughter tubers starting from the potato root system. Under field conditions, ISCI 4x-breeding clones performed extremely well, as did their male donor parent Q 115-6, which consistently demonstrated remarkable wireworm tolerance. Q 115-6 reported as low as 0% damage in several trials, indicating its strong potential as a wireworm-tolerant genotype. Similarly, both ISCI 181/10-3 and ISCI 201/10-1 showed low levels of total and severe damage, establishing their resilience across multiple regions and years.

When comparing total damage and severe damage, the trends for ISCI 4x-breeding clones remained consistent, with low percentages in both damage categories. This suggests that these genotypes are tolerant to minor damage, as well as to the more severe, yield-threatening impact of wireworms. Indeed, although the damage rates (%) of ISCI 133/12-7 and ISCI 181/10-4 varied across years and regions, they continuously outperformed many commercial varieties, particularly in the more severe damage assessments. On the other hand, commercial varieties, particularly Monalisa and Monique, consistently showed high wireworm damage levels. Monalisa was particularly vulnerable, with overall damage reaching up to 89.20% in 2018, and severe damage peaking at 56.80% the same year, as recently confirmed by Hurtado et al. [57]. Its consistently high susceptibility across both damage types suggests that Monalisa is highly susceptible, making it unsuitable for potato-producing basins prone to wireworm infestations, unless additional protective measures are implemented.

Regional data added another layer of insight. In Emilia-Romagna (Budrio), ISCI 4x-breeding clones generally showed better performance, with lower percentages of both total and severe damage than at other sites. In contrast, the commercial varieties Monalisa and Monique not only performed poorly in terms of total damage but also showed high levels of severe damage, indicating a high risk of significant crop loss in cultivating regions. Furthermore, Veneto and Lombardy reported some of the lowest percentages of severe damage for ISCI 4x-breeding clones, reinforcing the belief that they are suited to a diverse range of environmental conditions. Their consistent performance across regions further supports the potential of ISCI 4x-breeding clones for broader agricultural adoption, especially in areas with varying degrees of wireworm pressure.

Overall, ISCI 4x-breeding clones demonstrated a robust tolerance to wireworm attacks, both in terms of total and severe damage, making them highly promising candidates for wireworm-prone regions. In contrast, the commercial varieties Colomba, Monalisa, Monique, and Vivaldi, while widely cultivated, exhibited significant vulnerabilities that may compromise yield value and require more intensive defense management practices. The stark contrast in performance between these groups underscores the importance of selecting varieties that offer both resilience and consistency in wireworm-damaged areas.

As expected, most tolerant ISCI genotypes suffered the least wireworm scars and holes. The only exception was ISCI 201/10-1, which incurred the highest damage. The same breeding clones that proved to be PTM tolerant [33] were also found to be wireworm tolerant. A significant positive association between the α-chaconine and caffeic acid content under the periderm and PTM larval mortality was found [33]. This makes it likely that these secondary plant metabolites can reduce the damage by both Lepidoptera (PTM) and Coleoptera pests (wireworms). ISCI 181/10-3 and wild relative *S. chacoense* #GLKS30919# showed the lowest damage, while ISCI 207/11-2 and Q 115-6 showed the lowest number of holes. This demonstrates a huge potential for tolerant ISCI genotypes to lessen the wireworm impact on potato production, as well as the potential for *S. chacoense* #GLKS30919# to be used as a genetic source in breeding programs to create new tolerant varieties. Jonasson and Olsson [14] cited that glycoalkaloids, natural toxic compounds found in the Solanaceae family, play a crucial role in potato-tuber resistance.

Hurtado et al. [58] found that Monalisa, a highly susceptible genotype, was neither more nor less attractive to wireworms than less susceptible varieties based on choice trials with volatiles. Tuber appetibility appears to be a key factor. In fact, glycoalkaloids (TGA, α-chaconine and α-solanine) are bioactive molecules known for their insecticide properties [17,18]. See the breeding program for resistance to the CPB [30], mentioned in the introduction.

Skin and flesh secondary plant metabolites, such as α-chaconine and caffeic acid, are strongly related to PTM larval mortality, which may explain the massive reduction in the tuber-erosion rate. Plant phenolic compounds are also essential for plant defense against insects, with their function as adaptive traits developing as a chemical deterrent against herbivore insects [58]. These ISCI 4x-breeding clones also perform well agronomically (Table 9), enabling them to be exploited in various ways.

Table 10 provides a value for cultivation and use (VCU) assessment of the six ISCI 4x-breeding clones and highlights key characteristics, such as maturity, tuber shape, skin and flesh color, dry matter content, total yield, main defects, strengths, and market perspectives.

The assessment was supplied by the public body Council for Agricultural Research and Economics (CREA), which independently evaluates new varieties for registration on Italy’s National List [59] under the supervision of the Italian Ministry of Agriculture, Food Sovereignty and Forests.

CREA with its Research Centre for Plant Protection and Certification (CREA-DC) implements standard protocols established by EU legislation [60] which are based on a long-term comparison with widely used varieties (e.g., Agata, Agria, Monalisa, [61]). VCU covers the following agronomic characteristics:

- yield;
- factors in the physical environment (e.g., susceptibility to damage);
- resistance to harmful organisms (e.g., blackleg, common scab or leafroll);
- quality (e.g., crisping/French fry quality, sensory texture quality).

The texture evaluation (2018–2022)was based on quantitative descriptive analysis (QDA) by a trained sensory panel, using raw materials from the Budrio site (Emilia-Romagna) according to the standard procedures in ISO 11035 (sensorial parameters) and ISO 8586 (panel expert).

ISCI 133/12-7 stands out as a mid-season genotype with an oval-round shape and attractive appearance thanks to its smooth skin. Its sensory traits after boiling are also very positive. However, it has some weaknesses, such as tuber cracks and skin blemishes, due to its susceptibility to the stem canker and black scurf disease (*Rhizoctonia solani* Kuhn, SCD).

ISCI 181/10-3 is a late-maturing genotype with high yield potential, but suffers from significant aesthetic problems, such as strong netted skin and skin disorders, which greatly reduce appeal. Although it shows excellent yield potential and strong tolerance to the potato tuber moth, it is recommended for breeding purposes only due to poor marketability.

ISCI 181/10-4, a mid-to-late season genotype, shares similar characteristics with ISCI 181/10-3, e.g., good yield potential and tolerance to potato tuber moths, but its netted skin compromises appearance. This genotype is mainly intended for the unwashed-potato market, reflecting its limited suitability to specific niches.

ISCI 201/10-1 is distinguished by its mid-to-early maturity and high yield potential but is hindered by skin defects and poor appearance. Like ISCI 181/10-3, it is intended solely for breeding purposes and is not considered commercially suitable.

ISCI 207/11-2 and ISCI 232/12-1 are late-maturing genotypes, which, despite showing good yield potential and a high tuber set per plant, suffer from significant skin blemishes and thus poor appearance. Both genotypes are recommended for breeding purposes only and have no direct market potential.

ISCI 133/12-7 seems to be the most promising genotype for the ware potato market, as it has smooth skin and an attractive appearance, which are crucial for marketability. Its positive traits after boiling make it appealing to consumers. Although it has some weaknesses, including susceptibility to stem canker and black scurf, it is considered commercially suitable. The other tolerant ISCI genotypes, despite a high yield potential and positive traits, including pest tolerance, suffer from significant aesthetic issues, which massively reduce their marketability. Most of these genotypes should be recommended for breeding purposes only, rather than for direct commercial sale.

Table 11 presents the varieties’ overall ranking in terms of wireworm susceptibility. The tolerant ISCI genotypes in the first five rows consistently demonstrated superior performance across all criteria, particularly in terms of their wireworm tolerance and lower erosion percentages. This is reflected in their overall rankings, with ISCI 201/10-1 and ISCI 181/10-3 securing the top positions (1st and 2nd respectively). In contrast, commercial varieties, such as Monique and Monalisa, prop up the ranking (13th and 14th respectively), with significantly poorer showings in both wireworm attacks and erosion resistance.

The tolerant ISCI genotypes not only dominated the top spots in the overall ranking but also exhibited consistent resilience to wireworm attacks. ISCI 201/10-1 and ISCI 181/10-3 showed strong resilience (both ranked 2.5), which is crucial for minimizing crop damage and loss. They also boast excellent erosion rankings, with ISCI 201/10-1 having the lowest score, indicating minimal damage and highlighting its potential for high yield and marketability.

ISCI 201/10-1 stands out as the top performer across all criteria, consistently ranking first for the erosion percentage and maintaining a strong position in wireworm-attack resistance. This makes it an ideal candidate for breeding programs designed to enhance crop resilience. On the other hand, the commercial variety Monalisa, ranking last in both wireworm attacks and erosion, is the most susceptible, suggesting that it may require additional protective measures or may be less suitable for regions prone to these issues.

The number of plots and locations tested provides a measure of data reliability. The ISCI 4x-breeding clones, particularly ISCI 181/10-4 (31 plots, 10 locations) and ISCI 181/10-3 (27 plots, 9 locations), were tested across a larger number of plots and locations than most of the commercial varieties. This extensive testing adds robustness to the results, ensuring that the rankings of these ISCI 4x-breeding clones are reliable and not influenced by limited or localized conditions. There is a clear trend whereby genotypes tested in a greater number of plots and locations (e.g., ISCI 181/10-4 and ISCI 201/10-1) tend to have consistently higher rankings. Conversely, genotypes, such as ISCI 232/12-1, which were tested in fewer plots (3) and locations (1), have lower overall rankings (12th). This may indicate that limited testing can lead to less reliable performance data, possibly due to unaccounted-for environmental variables.

## 5. Conclusions

This long-term study shows that PTM-tolerant potato genotypes are tolerant to wireworm attacks as well. PTM mortality was significantly associated with phenolic and glycoalkaloid contents; it is thus likely that the effects on wireworms can be attributed to these compounds. Nevertheless, no significant increase in wireworm mortality was found when the larvae fed on the tubers (Table 6).

This implies that the use of PTM-tolerant genotypes as part of an IPM Prevention package has great potential against wireworms in potato crops. The susceptibility to wireworms of a significant sample of commercial and new breeding clones can be displayed practically, as in Table 11, which summarizes the results of field trials. It clearly shows that tolerant genotypes are available and that they may significantly reduce potato wireworm damage. This means that, depending on wireworm density, they can be used on their own, or as part of a wireworm package by potato growers, without synthetic insecticides being applied [62]. Our results suggest that the studied tolerant varieties are likely to work where *Agriotes* are the prevalent species and in similar environmental/biotic conditions. Their efficiency, however, should be further tested where other wireworm genera and climatic conditions are prevalent.

Once the risk of wireworm damage has been assessed [63,64] and monitoring with traps for click beetles [65] and larvae [37] has ascertained the wireworm-population density, a range of IPM packages can be implemented. When needed, further strategies can be added to provide tolerant genotypes with extra protection, e.g., biocidal cover crops [66,67], entomopathogens [68], naturally derived ingredients [34], plus other agronomic strategies, and tools [62].

This research outcome can therefore be exploited by the following:

- using the tolerant ISCI 4x-breeding clones or their donor parent Q 115-6 in breeding programs;
- reproducing the most agronomically promising genotypes (Table 10) to increase the amount of land farmed with tolerant potato varieties.

As a suggestion for decision-makers, it seems to be in the general interest to consider planting tolerant varieties in a bid to reduce farming costs and pollution while increasing potato health. This could be pursued by making it compulsory to:

- test tolerance to wireworm attacks in breeding programs, with a range of species and climatic conditions;
- publish yearly tables of variety susceptibility to wireworms, along with other agronomic characteristics based on independent public assessment (e.g., Table 11).

## Figures and Tables

**Table 1:** List of potato genotypes assessed in the pot trial.

Genotype	Dealer/Breeder/Maintainer
Agata	AGRICO, Emmeloord, The Netherlands
Ambra	HZPC, Joure, The Netherlands
Avanti	STET HOLLAND, Emmeloord, The Netherlands
Belami	IPM, Tincques, France
Colomba	HZPC, Joure, The Netherlands
ISCI 181/10-3	CREA-CI, Bologna, Italy
ISCI 181/10-4	CREA-CI, Bologna, Italy
ISCI 201/10-1	CREA-CI, Bologna, Italy
ISCI 207/11-2	CREA-CI, Bologna, Italy
Q 115-6	INIA, Osorno, Chile
JB007	BERNARD, Gomiècourt, France
Monalisa	HZPC, Joure, The Netherlands
Sensation	IPM, Tincques, France
*Solanum chacoense* #GLKS30919#	IPK-GLKS, Gross Luesewitz, Germany
Vivaldi	HZPC, Joure, The Netherlands

**Table 2:** Types of potato erosion.

Type of Erosion	Diameter (mm)	Characteristics
Small	1–2	Open wound
Ordinary	2–5	Open wound
Large	>5	Open wound
Old	Variable	Healed, deformed hole due to early attack and subsequent tuber development

**Table 3:** Main characteristics of field plot trials. All agronomic practices are given in the complete Table S1 as Supplementary Materials.

Region	Emilia- Romagna	Emilia- Romagna	Emilia- Romagna	Emilia- Romagna	Emilia- Romagna	Veneto	Veneto	Lombardy	Friuli- Venezia Giulia
Site	Budrio	Budrio	Budrio	Budrio	Budrio	Asigliano Veneto	Noventa Vicentina	Madesimo	Valvasone Arzene
Geographical coordinates	44.53630, 11.49303	44.53630, 11.49303	44.53630, 11.49303	44.53630, 11.49303	44.53630, 11.49303	45.30562, 11.44751	45.29612, 11.55413	46.25444, 9.20416	46.00559, 12.83533
Year	2018	2019	2020	2022	2023	2022	2023	2023	2023
Soil texture classification	Sandy clay loam	Sandy clay loam	Sandy clay loam	Sandy clay loam	Sandy clay loam	Clay loam	Clay loam	Sandy loam	Silty loam
Genotype	ISCI 181/10-3 ISCI 181/10-4 ISCI 201/10-1 ISCI 207/11-2 ISCI 232/12-1 Q 115-6 Bionica Monalisa Monique Morene	ISCI 181/10-3 ISCI 181/10-4 ISCI 201/10-1 ISCI 207/11-2 Q 115-6 Bionica Monalisa Monique Morene	ISCI 181/10-3 ISCI 181/10-4 ISCI 201/10-1 ISCI 207/11-2 Q 115-6 Bionica Monalisa Morene	ISCI 133/12-7 ISCI 181/10-3 ISCI 181/10-4 ISCI 201/10-1 ISCI 207/11-2 **Primura**	ISCI 133/12-7 ISCI 181/10-3 ISCI 181/10-4 **Agata** Vivaldi	ISCI 133/12-7 ISCI 181/10-4 ISCI 201/10-1 ISCI 207/11-2 **Primura**	ISCI 181/10-3 ISCI 181/10-4 ISCI 133/12-7 **Agata** Vivaldi	ISCI 133/12-7 ISCI 181/10-3 ISCI 181/10-4 Monique	ISCI 181/10-3 ISCI 181/10-4 **Agata** Vivaldi
Seed spacing (cm)	90*30	90*30	90*30	90*30	90*30	80*27	90*24	80*24	80*28
Tuber-seed size (Ø mm), no cutted	45–55	45–55	45–55	45–55	45–55	35–45	34–45	35–45	35–50
Planting date	7 March	3 March	9 March	5 March	6 March	12 March	8 March	6 June	30 March
Harvest date	21 August	19 August	21 August	20 July (Primura) 14 August	14 July (Agata) 22 August	03 August	11 >August	22 October	17 August

Genotypes with early maturity are in bold.

**Table 4:** Pot trials. The table presents the median values of “Total erosions” and “Severe erosions” for different potato genotypes. The letters next to the median values indicate groups of genotypes that are not significantly different from each other, according to Tukey’s HSD test (*p* < 0.05). The original data were transformed into ranks before conducting the ANOVA. Genotypes with the same letter belong to the same statistical group.

Genotype	Total Erosions		Severe Erosions	
Colomba	7.5	a	7	a
Ambra	5.5	ab	5	ab
Monalisa	5.5	abc	5	ab
JB 007	5.5	ab	4.5	ab
Sensation	5	abc	4	ab
Vivaldi	4.5	abc	4.5	ab
Avanti	4	abc	2.5	ab
ISCI 201/10-1	3.5	abc	3	ab
ISCI 181/10-4	3	abc	3	ab
Agata	3.5	abc	2	ab
Belami	3	abc	2.5	ab
Q 115-6	2.5	bc	2	b
ISCI 181/10-3	1.5	bc	1	b
ISCI 207/11-2	1.5	bc	1	b
*Solanum chacoense* #GLKS30919#	1	c	1	b

The probability level was assessed using ANOVA on ranked data. The separation of rank means was performed using the Tukey HSD test (*p* < 0.05).

**Table 5:** Comparison between the variety Colomba and 14 other commercial varieties/breeding clones/wild species accession, showing the probabilities of statistical differences for both “Total erosions” and “Severe damage”. Colomba was selected as the benchmark since it scored the highest number of wireworm erosions (Table 4). The values in the two columns indicate the probability (%) that no significant differences exist between Colomba and each of the other genotypes. The colors range from green (high probability of significant differences) to red (low probability).

Genotype Comparison		Probability (%)	
		Total Erosions	Severe Damage
Colomba vs.	*Solanum chacoense* #GLKS30919#	0.10	0.27
	ISCI 181/10-3	0.82	1.48
	Q 115-6	2.44	1.61
	ISCI 207/11-2	3.32	3.72
	ISCI 201/10-1	24.92	20.90
	Belami	38.11	21.79
	Agata	44.65	19.46
	ISCI 181/10-4	32.06	35.42
	Avanti	61.84	40.95
	Sensation	98.25	98.29
	Monalisa	98.64	99.73
	Ambra	99.98	99.47
	Vivaldi	99.53	99.97
	JB 007	99.68	99.91

The probability level was assessed using ANOVA on ranked data.

**Table 6:** Survival of larvae in pots. Medians of larvae retrieved from the trial or missing. The probability level was assessed using ANOVA on ranked data. The separation of rank means was performed using the Tukey HSD test (*p* < 0.05).

Genotype	% Alive Larvae		% Dying Larvae		% Dead Larvae		% Missing Larvae	
Agata	83.3		0.0	a	0.0		16.7	
Ambra	91.7		0.0	a	0.0		8.3	
Avanti	66.7		0.0	a	0.0		33.3	
Belami	83.3		0.0	a	0.0		16.7	
Colomba	75.0		0.0	a	0.0		25.0	
ISCI 181/10-3	100.0		0.0	a	0.0		0.0	
ISCI 181/10-4	75.0		0.0	a	0.0		25.0	
ISCI 201/10-1	75.0		0.0	a	0.0		25.0	
ISCI 207/11-2	91.7		0.0	a	0.0		8.3	
Q 115-6	83.3	a	0.0	a	0.0		16.7	
JB 007	91.7	a	0.0	a	0.0		8.3	
Monalisa	83.3	a	0.0	a	0.0		16.7	
Sensation	83.3	a	0.0	a	0.0		16.7	
*Solanum chacoense* #GLKS30919#	91.7	a	0.0	a	0.0		8.3	
Vivaldi	91.7	a	0.0	a	0.0		0.0	
**Sign.**	NS		NS		NS		NS	
**P**	0.5896		0.1772		0.6774		0.5008	
**DoF**	89		89		89		89	

The probability level was assessed using ANOVA on ranked data. The separation of rank means was performed using the Tukey HSD test (*p* < 0.05). “Sign.” stands for level of Significance; “P” stands for Probability; “DoF” stands for degree of freedom.

**Table 7:** Wireworms caught by bait traps or found in damaged tubers potatoes at harvest in the studied fields. n.a. = not assessed.

Year	Region	Site	Wireworm Species	
			No./Bait Trap	No. in Damaged Potatoes
2018	Emilia-Romagna	Budrio	n.a.	5, *A. sordidus*
2019	Emilia-Romagna	Budrio	n.a.	3, *A. sordidus*
2020	Emilia-Romagna	Budrio	n.a.	0
2022	Emilia-Romagna	Budrio	n.a.	0
2022	Veneto	Asigliano Veneto	0.10 *A. sordidus*	1, *A. sordidus*
2023	Emilia-Romagna	Budrio	1.11 *A. sordidus*	2, *A. sordidus*
2023	Veneto	Noventa Vicentina	n.a.	2, *A. sordidus*
2023	Lombardy	Madesimo	n.a.	0
2023	Friuli-Venezia Giulia	Valvasone Arzene	n.a.	1, *A. brevis*

**Table 8:** Percentage of tubers with wireworm damage (median). The original data were transformed into ranks before conducting the ANOVA. Genotypes with the same letter belong to the same statistical group.

Region	Emilia- Romagna		Emilia- Romagna		Emilia- Romagna		Emilia- Romagna		Emilia- Romagna		Veneto		Veneto		Lombardy		Friuli- Venezia Giulia	
Site	Budrio		Budrio		Budrio		Budrio		Budrio		Asigliano Veneto		Noventa Vicentina		Madesimo		Valvasone Arzene	
Q 115-6	26.40	c	5.36	c	0.00	c												
ISCI 133/12-7							7.83	ab	23.90	bc	4.31	bc	4.35	b	0.00	c		
ISCI 181/10-3	26.60	c	6.36	c	1.79	bc	3.45	b	15.00	c			1.65	c	0.00	c	3.14	ab
ISCI 181/10-4	44.30	abc	9.35	bc	5.83	a	5.79	ab	22.20	bc	5.47	ab	4.72	b	0.96	b	2.19	b
ISCI 201/10-1	26.40	c	7.41	c	0.98	c	2.52	b			1.87	c						
ISCI 207/11-2	46.80	abc	10.00	bc	4.76	ab	4.92	ab			2.20	c						
ISCI 232/12-1	56.70	abc																
Agata									86.40	a			8.43	a			4.38	ab
Bionica	40.20	abc	9.02	bc	0.92	c												
Monalisa	89.20	a	41.50	a	22.50	a												
Monique	61.00	ab	24.80	ab											9.00	a		
Morene	64.30	ab	13.00	bc	4.23	ab												
Primura							14.70	a			8.62	a						
Vivaldi									78.70	ab			16.50	a			7.67	a

The probability level was assessed using ANOVA on ranked data. The separation of rank means was performed using the Tukey HSD test (*p* < 0.05).

**Table 9:** Percentage of tubers (median) with severe wireworm damage (at least one ordinary or big erosion/hole). The original data were transformed into ranks before conducting the ANOVA. Genotypes with the same letter belong to the same statistical group.

Region	Emilia- Romagna		Emilia- Romagna		Emilia- Romagna		Emilia- Romagna		Emilia- Romagna		Veneto		Veneto		Lombardy		Friuli- Venezia Giulia	
Site	Budrio		Budrio		Budrio		Budrio		Budrio		Asigliano Veneto		Noventa Vicentina		Madesimo		Valvasone Arzene	
Q 115-6	16.10	bc	1.82	c	0.00	c												
ISCI 133/12-7							5.22	ab	20.70	bc	4.02	ab	3.62	bc	0.00	B		
ISCI 181/10-3	21.50	abc	4.42	c	0.93	bc	4.42	ab	10.20	c			0.75	c	0.00	B	2.02	ab
ISCI 181/10-4	11.30	c	8.11	c	2.91	a	5.79	ab	18.10	bc	3.95	ab	3.77	bc	0.00	B	0.58	b
ISCI 201/10-1	11.20	bc	6.48	bc	0.00	a	1.68	b			0.90	b						
ISCI 207/11-2	33.90	abc	7.27	c	2.86	ab	0.86	b			2.20	ab						
ISCI 232/12-1	43.30	ab																
Agata									83.90	a			5.08	ab			2.35	ab
Bionica	34.50	abc	5.74	bc	0.92	bc												
Monalisa	56.80	a	31.10	a	15.30	a												
Monique	48.80	ab	20.00	ab											7.00	A		
Morene	48.80	abc	5.41	c	2.82	ab												
Primura							20.20	a			5.98	a						
Vivaldi									64.80	ab			13.40	a			5.07	a

Probability was assessed using ANOVA on ranked data. The separation of rank means was performed using the Tukey HSD test (*p* < 0.05).

**Table 10:** Main agronomic and morpho-physiological traits of ISCI’s potato 4x-breeding clones evaluated during field trials compared with the commercial variety Colomba.

Genotype	Maturity	Tuber Shape	Skin and Flesh Color	Tuber Dry Matter Content (%)	Total Yield Range (t ha^−1^)	Weaknesses	EAPR Cooking Type	Strengths	Market End-Use and Perspectives
Colomba	early	round oval-oval	yellow/ yellow	16.5–17.5	50–60	sensibility to second growth and sprouting, PVY	B (suitable for multiple uses)	very good tuber appearance, good taste and texture	early and ware potatoes
ISCI 133/12-7	mid-late	oval-round	yellow/ yellow	20.5–21.5	45–50	rhizo cracks, PVYand stem-end rot	AB (suitable for salad and multiple uses)	good tuber appearance, taste and texture	ware potatoes
ISCI 181/10-3	late to very late	oval-round	yellow/ yellow	21.5–22.5	50–55	strong netted skin, skin disorders and blemishes, tuber bruising	BC (suitable for home fries)	high tolerance to potato tuber moth	for breeding use only, high pollen fertility
ISCI 181/10-4	mid-late to late	oval-round	yellow/ yellow	18.5–19.5	45–50	netted skin, misshapen tubers	BC (suitable for home fries)	high tolerance to potato tuber moth	ware potatoes, for the market of unwashed potatoes only
ISCI 201/10-1	mid-early	round-oval	yellow/white	16.5–17.5	50–55	skin disorders and blemishes	AB (suitable for salad and multiple uses)	high tolerance to potato tuber moth	for breeding use only, high pollen fertility
ISCI 207/11-2	mid-late to late	oval-round	yellow/ yellow	18.5–19.5	45–50	netted skin, severe after cooking blackening	B (suitable for multiple uses)	high tuber set per plant	for breeding use only, high pollen fertility
ISCI 232/12-1	mid-late to late	round-oval	yellow/white	17.5–18.5	50–55	netted skin, skin, disorders and blemishes	B (suitable for multiple uses)	high total yield potential	for breeding use only, high pollen fertility

**Table 11:** Results by genotype, including the number of plots, locations and rankings for wireworm attacks, and the percentage of ordinary and large erosion rates. The “Overall ranking” column presents the genotypes in terms of total wireworm damage, from the best, i.e., the least damage (ISCI 201/10-1) to the worst, i.e., the most damaged (Monalisa). In the first two columns, darker blue shades indicate higher values for the number of plots and locations, with lighter shades representing lower numbers. In the next three columns, dark green marks the top-ranking genotypes, yellow intermediate positions, and red the lowest.

Variety	Number of Plots	Number of Locations	Ranking Wireworm Attacks	Ranking Percentage Erosion Ordinary + Large	Total Ranking
ISCI 201/10-1	15	5	2.5	1	1
ISCI 181/10-3	27	9	2.5	2	2.5
Q 115-6	9	3	1	3	2.5
ISCI 133/12-7	16	5	4	4	4
ISCI 181/10-4	31	10	5	5	5
Agata	12	4	6	6	6
ISCI 207/11-2	16	5	7	7.5	7
Vivaldi	12	4	8.5	7.5	8
Bionica	9	3	8.5	9	9
Morene	9	3	11	10	10
Primura	8	2	10	11	11
ISCI 232/12-1	3	1	12	13	12
Monique	9	3	13	12	13
Monalisa	9	3	14	14	14

## Data Availability

The original contributions presented in the study are included in the article/Supplementary Materials, further inquiries can be directed to the corresponding author.

## Supplementary Materials

The following supporting information can be downloaded at: https://www.mdpi.com/article/10.3390/insects16010004/s1, Table S1. Main agronomic and experimental characteristics of field plot trials.
