# Supplementary material for: Integrated Pest Management of Wireworms in Potatoes: Use of Tolerant Varieties to Implement Damage Prevention
Source: Insects. 2024 Dec 26;16(1):4. doi: 10.3390/insects16010004 (PMC11765998; doi:10.3390/insects16010004)
Supplement: Supplementary file 1 [file insects-16-00004-s001.zip › insects-3252228-supplementary.pdf]

Table S1. Main agronomic and experimental characteristics of field plot trials.

Administrative region	Emilia-Romagna	Emilia-Romagna	Emilia-Romagna	Emilia-Romagna	Emilia-Romagna	Veneto	Veneto	Lombardy	Friuli-Venezia Giulia
Municipality	Budrio	Budrio	Budrio	Budrio	Budrio	Asigliano Veneto	Noventa Vicentina	Madesimo	Valvasone Arzene
Geographical coordinates	44.53630, 11.49303	44.53630, 11.49303	44.53630, 11.49303	44.53630, 11.49303	44.53630, 11.49303	45.30562, 11.44751	45.29612, 11.55413	46.25444, 9.20416	46.00559 12.83533
Year	2018	2019	2020	2022	2023	2022	2023	2023	2023
Soil texture classification	Sandy clay loam	Sandy clay loam	Sandy clay loam	Sandy clay loam	Sandy clay loam	Clay loam	Clay loam	Sandy loam	Silty loam
Replications (no.)	3	3	3	3	3	3	3	3	3
Tillage operations prior to potato planting	Fall ploughing, harrowing and preplant bedding	Fall ploughing, harrowing and preplant bedding	Fall ploughing, harrowing and preplant bedding	Fall ploughing, harrowing and preplant bedding	Fall ploughing, harrowing and preplant bedding	Spring ploughing and harrowing	Spring subsoiling, ploughing and harrowing	Spring ploughing and harrowing	Spring subsoiling, ploughing and harrowing
Preceding crops (3y-based)	Sugarbeet → Sunflower → Durum wheat	Maize→ Sugarbeet → Durum wheat	Winter barley→ Grain sorghum→ Durum wheat	Maize→ Sunflower → Durum wheat	Sugarbeet → Grain sorghum→ Durum wheat	Soybean→ Maize→ Brown mustard green manure	Soft wheat→ Maize→ Brown mustard green manure	Long-term mountain meadow → Potato	Soybean→ Maize→ Grafted vines nursery
Genotype	ISCI 181/10-3<br>ISCI 181/10-4<br>ISCI 201/10-1<br>ISCI 207/11-2<br>ISCI 232/12-1<br>Q 115-6<br>Bionica<br>Monalisa<br>Monique<br>Morene	ISCI 181/10-3<br>ISCI 181/10-4<br>ISCI 201/10-1<br>ISCI 207/11-2<br>Q 115-6<br>Bionica<br>Monalisa<br>Monique<br>Morene	ISCI 181/10-3<br>ISCI 181/10-4<br>ISCI 201/10-1<br>ISCI 207/11-2<br>Q 115-6<br>Bionica<br>Monalisa<br>Morene	ISCI 133/12-7<br>ISCI 181/10-3<br>ISCI 181/10-4<br>ISCI 201/10-1<br>ISCI 207/11-2<br>Primura	ISCI 133/12-7<br>ISCI 181/10-3<br>ISCI 181/10-4<br>Agata<br>Vivaldi	ISCI 133/12-7<br>ISCI 181/10-4<br>ISCI 201/10-1<br>ISCI 207/11-2<br>Primura	ISCI 181/10-3<br>ISCI 181/10-4<br>ISCI 133/12-7<br>Agata<br>Vivaldi	ISCI 133/12-7<br>ISCI 181/10-3<br>ISCI 181/10-4<br>Monique	ISCI 181/10-3<br>ISCI 181/10-4<br>Agata<br>Vivaldi
Seed spacing (cm)	90*30	90*30	90*30	90*30	90*30	80*27	90*24	80*24	80*28
Tuber-seed size (Ø mm), not cut	45–55	45–55	45–55	45–55	45–55	35–45	34–45	35–45	35–50
Planting date	March 7	March 3	March 9	March 5	March 6	March 12	March 8	June 6	March 30
Chemical herbicide after row-ridging	(Stomp Aqua, Basf) + *aclonifen* (Challenge, Bayer CropScience) + *flufenacet* and *metribuzin* (Fedor, Bayer Cropscience)	*pendimetalin* (Stomp Aqua, Basf) + *aclonifen* (Challenge, Bayer CropScience) + *flufenacet* and *metribuzin* (Fedor, Bayer Cropscience)	*pendimetalin* (Stomp Aqua, Basf) + *aclonifen* (Challenge, Bayer CropScience) + *flufenacet* and *metribuzin* (Fedor, Bayer Cropscience)	*pendimetalin* (Stomp Aqua, Basf) + *aclonifen* (Challenge, Bayer CropScience) + *flufenacet* and *metribuzin* (Fedor, Bayer Cropscience)	*pendimetalin* (Stomp Aqua, Basf) + *aclonifen* (Challenge, Bayer CropScience) + *flufenacet* and *metribuzin* (Fedor, Bayer Cropscience)	*pendimetalin* (Stomp Aqua, Basf) + *aclonifen* (Challenge, Bayer CropScience) + *metribuzin* (Feinzin 70 DF, Adama) + *clomazone* (Sirtaki, Sipcam)	*pendimetalin* (Stomp Aqua, Basf) + *aclonifen* (Challenge, Bayer CropScienc e) + *clomazone* (Sirtaki, Sipcam)	No treatment	*pendimetalin* (Stomp Aqua, Basf) + *aclonifen* (Challenge, Bayer CropScience) + *clomazone* (Clematis, Adama) + *napropamide* (Devrinol, UPL)
Chemical herbicide after row-ridging	No treatment	No treatment	No treatment	No treatment	No treatment	No treatment	No treatment	No treatment	*rimsulfuron* (Executive, Corteva)
Mineral fertilization at planting (kg/ha of N, P_2O_5, K_2O, MgO)	140 (N)<br>185 (P_2O_5)<br>199 (K_2O)<br>28 (MgO)	140 (N)<br>185 (P_2O_5)<br>199 (K_2O)<br>28 (MgO)	140 (N)<br>185 (P_2O_5)<br>199 (K_2O)<br>28 (MgO)	140 (N)<br>185 (P_2O_5)<br>199 (K_2O)<br>28 (MgO)	140 (N)<br>185 (P_2O_5)<br>199 (K_2O)<br>28 (MgO)	96 (N)<br>288 (P_2O_5)<br>288 (K_2O)	45 (N)<br>115 (P_2O_5)<br>300 (K_2O)	No chemical fertilizatio n	32 (N)<br>36 (P_2O_5)<br>162 (K_2O)
Mineral fertilization at row ridging (kg/ha of N, K_2O, MgO)	60 (N)<br>100 (K_2O)<br>33 (MgO)	60 (N)<br>100 (K_2O)<br>33 (MgO)	60 (N)<br>100 (K_2O)<br>33 (MgO)	60 (N)<br>100 (K_2O)<br>33 (MgO)	60 (N)<br>100 (K_2O)<br>33 (MgO)	210 (N)	175 (N)	No chemical fertilizatio n	By fertigation<br>42 (N)<br>43 (P_2O_5)<br>34 (K_2O)

Irrigation systems	Spray boom	Spray boom	Spray boom	Spray boom	Spray boom	Solid-set sprinklers	Hose reel	No irrigation (1560 m asl)	Drip irrigation
Disease chemical control (active ingredient /commercial products/no. of foliar sprayings)	*copper hydroxide* + *copper oxychloride* (Grifon 280, Gowan) x 2; *fluopicolide* + *propamocarb* (Volare, Bayer CropScience) x 3; *fluazinam* (Nando Maxi, Sumitomo) + *difeconazole* (Score 25 EC, Syngenta) x 2	*copper hydroxide* + *copper oxychloride* (Grifon 280, Gowan) x 2; *fluopicolide* + *propamocarb* (Volare, Bayer CropScience) x 3; *fluazinam* (Nando Maxi, Sumitomo) + *difeconazole* (Score 25 EC, Syngenta) x 2	*copper hydroxide* + *copper oxychloride* (Grifon 280, Gowan) x 2; *fluopicolide* + *propamocarb* (Volare, Bayer CropScience) x 3; *fluazinam* (Nando Maxi, Sumitomo) + *difeconazole* (Score 25 EC, Syngenta) x 2	*copper hydroxide* + *copper oxychloride* (Grifon 280, Gowan) x 2; *fluopicolide* + *propamocarb* (Volare, Bayer CropScience) x 3; *fluazinam* (Nando Maxi, Sumitomo) + *difeconazole* (Score 25 EC, Syngenta) x 2	*copper hydroxide* + *copper oxychloride* (Grifon 280, Gowan) x 2; *fluopicolide* + *propamocarb* (Volare, Bayer CropScience) x 3; *fluazinam* (Nando Maxi, Sumitomo) + *difeconazole* (Score 25 EC, Syngenta) x 2	*metalaxyl* + *copper sulfate* (Ridomil Gold R, Syngenta), x 3; *fluopicolide* + *propamocarb* (Volare, Bayer CropScience), x 2 + *difeconazole* (Score 25 EC, Syngenta), x 3	*cimoxanil* (Carson 45 WG, Adama) + *fluazinam* (Ohayo, Certis Belchim); *oxathiapiprolin* (Zorvec Epicaltrin, Corteva) x 3; *cimoxanil* (Carson 45 WG, Adama) + *copper sulfate* (Ridomil Gold R, Syngenta); *cimoxanil* (Carson 45 WG, Adama) + *zoxamide* (Zoxium 240 SC, Gowan)	No treatment	*metiram* (Polyram DF, Basf) + *fluazinam* (Banjo, Adama); *cyazofamid* (Canthico, Corteva) + *cimoxanil* (Vitene Ultra SC, Sipcam); *cyazofamid* (Azuleo, Adama) + *oxathiapiprolin* (Zorvec Epicaltrin, Corteva) + *difeconazolo* (Mavita 250 EC, Adama); *propamocarb* + *cimoxanil* (Axidor, Chimiberg) + *zoxamide* (Zoxium 240 SC, Gowan); *metalaxyl* + *copper sulfate* (Ridomil Gold R, Syngenta) + *difeconazole* (Score 25 EC, Syngenta); *zoxamide* + *cimoxanil* (Lieto SC, Sipcam) + *copper sulphate* (Cupravit Bio Advanced, Bayer CropScience); *dimetomorf* + *pyraclostrobin* (Cabrio Duo, Basf)
Pest chemical control (active ingredient /commercial products/no. of foliar sprayings)	*acetamiprid* (Kestrel, Sumitomo) x 2; *chlorantraniliprole* (Voliam, Syngenta) x 2	*acetamiprid* (Kestrel, Sumitomo) x 2; *chlorantraniliprole* (Voliam, Syngenta) x 2	*acetamiprid* (Kestrel, Sumitomo) x 2; *chlorantraniliprole* (Voliam, Syngenta) x 2	*acetamiprid* (Kestrel, Sumitomo) x 2; *chlorantraniliprole* (Voliam, Syngenta) x 2	*acetamiprid* (Kestrel, Sumitomo) x 2; *chlorantraniliprole* (Voliam, Syngenta) x 2	*acetamiprid* (Epik SL, Sipcam) x 2; *chlorantraniliprole* (Coragen, FMC) x 2	*acetamiprid* (Epik SL, Sipcam); *metaflumizone* (Alverde, BASF)	No treatment	*lambda-cyalotrin* (Sparviero, Sipcam); *acetamiprid* (Epik SL, Sipcam)

Haulm killing after foliage removal by chopper beater	*carfrentazone-ethyl* (Spotlight, FMC)	*carfrentazone-ethyl* (Spotlight, FMC)	*carfrentazone-ethyl* (Spotlight, FMC)	*carfrentazone-ethyl* (Spotlight, FMC)	*carfrentazone-ethyl* (Spotlight, FMC)	No treatment	No treatment	No treatment	*carfrentazone-ethyl* (Spotlight, FMC)
Harvest date	August 21	August 19	August 21	July 20 (Primura) August 14	July 14 (Agata) August 22	August 3	August 11	October 22	August 17
